# Dynamic Influence of Emotional States on Novel Word Learning

**DOI:** 10.3389/fpsyg.2018.00537

**Published:** 2018-04-11

**Authors:** Jingjing Guo, Tiantian Zou, Danling Peng

**Affiliations:** ^1^Shaanxi Key Laboratory of Behavior and Cognitive Neuroscience, School of Psychology, Shaanxi Normal University, Xi'an, China; ^2^State Key Laboratory of Cognitive Neuroscience and Learning, Beijing Normal University, Beijing, China

**Keywords:** word learning, emotional modulation, dynamic, positive, negative

## Abstract

Many researchers realize that it's unrealistic to isolate language learning and processing from emotions. However, few studies on language learning have taken emotions into consideration so far, so that the probable influences of emotions on language learning are unclear. The current study thereby aimed to examine the effects of emotional states on novel word learning and their dynamic changes with learning continuing and task varying. Positive, negative or neutral pictures were employed to induce a given emotional state, and then participants learned the novel words through association with line-drawing pictures in four successive learning phases. At the end of each learning phase, participants were instructed to fulfill a semantic category judgment task (in Experiment 1) or a word-picture semantic consistency judgment task (in Experiment 2) to explore the effects of emotional states on different depths of word learning. Converging results demonstrated that negative emotional state led to worse performance compared with neutral condition; however, how positive emotional state affected learning varied with learning task. Specifically, a facilitative role of positive emotional state in semantic category learning was observed but disappeared in word specific meaning learning. Moreover, the emotional modulation on novel word learning was quite dynamic and changeable with learning continuing, and the final attainment of the learned words tended to be similar under different emotional states. The findings suggest that the impact of emotion can be offset when novel words became more and more familiar and a part of existent lexicon.

## Introduction

Word learning is an important cognitive process for individuals to master knowledge and adapt to modern society. Moreover, learning new words is a necessary first step in learning a new language (Lesage et al., 2016). Numerous studies have been investigating the neurocognitive mechanisms of word learning (Nielson and Bryant, 2005; Mestres-Missé et al., 2014; Zhao et al., 2014), but few studies considered the regulating effect of emotion on word learning, which was in stark contrast to the research trend toward exploring the interactions between cognition and emotion systems. Considerable research has investigated the role which emotion plays in numerous aspects of cognition, including memory (Brunyé et al., 2009; Baddeley, 2013), cognitive flexibility (Van Wouwe et al., 2011), problem solving (Isen et al., 1987), judgment and decision making (Isen, 1993; Schwarz, 2000; Blanchette and Richards, 2010), complex learning (D'mello and Graesser, 2012), reasoning (Ifenthaler, 2015), multitasking (Morgan and D'mello, 2016), and multimedia learning (Um and Plass, 2012; Park et al., 2014, 2015; Plass et al., 2014; Knörzer et al., 2016). However, whether emotion inhibits or facilitates successful word learning has been elusive. Therefore, the present study aims to explore the mechanism of novel word learning under emotional modulation.

The evidence is accumulating to demonstrate the important role of emotion in word processing (Olafson and Ferraro, 2001; Nakic et al., 2006; Wang et al., 2014). Plenty of research indicated that the more arousing a word is, the faster this word is recognized (Larsen et al., 2008; Hofmann et al., 2009). For instance, lexical decision for negative words was faster than that for neutral words and increased the activation in bilateral amygdala and middle temporal cortex. It was argued that neural activity of amygdala induced by negative arousal may facilitate the retrieval and selection of semantic representation (Nakic et al., 2006). The emotionality of words not only impacts word processing *per se*, but also can affect the processing of other stimuli. For example, recent studies found that emotional words could facilitate the categorization of emotional picture (Liu et al., 2010) and even changed the preference to novel symbols(Guo et al., 2011). These findings agree with the model proposed by Peng et al. (2006), which suggested that emotional brain areas such as amygdala modulated the activation of anterior and posterior neural networks involved in words' grapheme, phonology and semantic processing (Pugh et al., 2000).

Notably, most of previous studies focused on modulation of emotion on existing words' processing rather than novel words' learning. Existing words' processing is different from novel words' learning in that learning necessitates dynamic developing process during which new associations between word symbol and semantic representation are established. Prior research has shown that novel words could be acquired by adults and integrated into their existing lexicon through a brief training period (Breitenstein et al., 2007; Mestresmissé et al., 2007; Mestres-Missé et al., 2014; Batterink and Neville, 2011; Borovsky et al., 2013; Tamminen and Gaskell, 2013; Chen et al., 2014). For example, Breitenstein et al. (2007) trained participants to learn the associations between novel spoken words and object pictures unintentionally for 5 consecutive days. The results of cross-modal semantic priming tests administered prior to and after the vocabulary training revealed that learned novel words' priming effects became similar to existing related words, and suggested that integrating the novel words into existing conceptual and lexical networks might occur after a 5-day training. However, without taking emotional modulation into account, aforementioned studies were not sufficient to elucidate the exact mechanism of novel word learning. Therefore, it's still a mystery how emotion modulates the process of novel word learning.

Although lack of direct evidence for the mechanism of novel word learning under emotional modulation, there was some research exploring the role of emotion in memorizing words or pictures. It was found that emotional arousal during encoding phase could boost the subsequent recognition or retrieval for learned words or pictures (Erk et al., 2005; Callan and Schweighofer, 2008; Ritchey et al., 2008). Furthermore, a robust enhancement effect of post-learning negative emotion (digest, sadness and anger) on consolidation of item memory was found but not for source memory (Nielson et al., 2005; Wang and Fu, 2010; Wang, 2015). These findings suggested that emotions could influence all the processes of word learning, including encoding, consolidation, recognition or retrieval.

When taking emotion into consideration, we cannot ignore that emotion consists of two dimensions: arousal and valence. There were a bunch of studies demonstrating that valence (ranging from negative to positive) and arousal (ranging from calming to exciting) exerted divergent effects on cognitive processing and that they differentiated in related neural substrates (Mourão-Miranda et al., 2003; Anders et al., 2004; Kensinger, 2004; Kensinger et al., 2004; Kensinger and Schacter, 2006). While some researchers only emphasized the role of arousal in the emotional memory enhancement (Hamann, 2001; Phelps, 2006; Mather, 2007), others found that valence played an important role in recognition memory (Adelman and Estes, 2013). However, some studies investigating the influence of emotions on learning didn't take negative emotions into account (Um and Plass, 2012; Plass et al., 2014; Park et al., 2015). Therefore, to better understand how different emotional states impact novel word learning, we included negative, positive and neutral emotional states, with comparable arousal between positive and negative conditions.

In the present study, participants tried to learn novel words after emotional states were induced by emotional pictures in the encoding phase. Emotion induction during learning may influence the attention allocation, encoding and consolidation for novel words' lexical semantics (Nielson and Arentsen, 2012). Since negative emotion often led to a narrowed attention focus (Fredrickson, 2000; Kaspar and König, 2012) and could distract learners from the learning materials (Seibert and Ellis, 1991), negative emotional state might impair attention allocation efficiency and thus result in worse learning performance. Moreover, according to Zeng et al. (2016), emotion-laden but task-irrelevant stimulus of a high threat impairs task performance. Therefore, we expected that negative emotional state would probably pose a suppressing effect on word learning.

In addition, negative and positive emotions seemed to have opposing effects on perception (Schmitz et al., 2009) and memory (Murty et al., 2011). Words evaluated more positively appeared to be recognized faster (Larsen et al., 2008; Kousta et al., 2009; Briesemeister et al., 2011), while negative words were often found to be processed slower than neutral ones (Kuchinke et al., 2005; Larsen et al., 2008; Hofmann et al., 2009). A beneficial effect was often found for positive emotions on cognitive processes and learning (Um and Plass, 2012; Plass et al., 2014; Ifenthaler, 2015; Park et al., 2015; Morgan and D'mello, 2016). For example, an eye-tracking study by Park et al. (2015) found that positive emotional state before learning led to better learning outcomes in comprehension and transfer tests and showed longer fixation durations on the text information of the learning environment. Hence, we expected that positive emotional state might facilitate word learning.

It's notable that previous studies tended to examine the effects of emotions on learning through comparing the performance after learning with the performance before learning, without few considerations for the dynamic learning process. So the present study shall try to investigate whether the effects of emotional states keep stable across the whole learning process or changeable from the beginning to the end of learning. In order to catch the dynamic modulation process, we asked participants to learn novel Hangul words through four learning phases and kept track of the effects of emotional states in each learning phase. We hypothesized that the effects of emotional states might be stronger in the early phases of learning. It is well-accepted that our adaptive body would spontaneously employ emotion regulation including extrinsic and intrinsic processes implied in monitoring, evaluating and modifying emotions (Thompson, 1994) when encountering emotional stimuli. And emotion regulation tends to decrease negative experience as well as increase or maintain positive affect (Carstensen et al., 2000) when emotion occurs at an inappropriate time or intense level. For example, healthy individuals can voluntarily regulate their negative emotional responses and successfully suppress negative feelings that can lead to decreased physiological activity and more intense negative effects (Ochsner et al., 2002, 2004; Anderson et al., 2004). It could be inferred that with learning continuing, emotion regulation might neutralize the emotional impact of the arousing stimuli (Richards, 2004), thereby narrowing the gap between impacts of positive and negative emotional states on novel word learning. Therefore, the effects of emotional states might be stronger in the early phases of learning and weakened then.

Besides, with the different levels of word learning tasks, the emotional states' modulating effects may differentiate. According to the well-known Yerkes-Dodson Law (Yerkes and Dodson, 1908), there is a negative quadratic relationship between arousal and performance (the “inverted-U” hypothesis), and the optimal level of arousal for a more difficult task will be lower than that for an easier task. Therefore, two learning tasks were employed in the current study, namely semantic category learning task and word specific meaning learning task. The former is easier than the latter since participants only need to grasp categorical information of the novel words in the former task while they have to obtain the one-to-one mapping between the novel words and their meanings in the latter task. We expected that the modulating effects of emotional states would be moderated by depth of learning tasks.

## Experiment 1: emotional modulation on novel words' semantic category learning

### Methods

#### Participants

Twenty Chinese students (6 males, aged from 18 to 22 years) from Beijing Normal University were paid to participate in the present experiment. All of them are right-handed and have normal or corrected to normal eyesight. All participants were screened by the Positive Affect and Negative Affect Scales (PANAS, Watson et al., 1988; Huang et al., 2003) to make sure that they were emotionally stable in recent weeks. All of them had no experience of learning Korean.

#### Materials

Korean Hangul words were chosen as the novel words since they have the similar square word form as the Chinese characters and their novelty to participants is easy to manipulate. 30 Hangul single words were selected and each word was paired with a dark and white line drawing picture which has a concrete animate or inanimate meaning. Examples for Hangul words were shown in Appendix A. Parameters of all pictures were matched, such as visual complexity (2.84 ± 0.43), familiarity (4.67 ± 0.16), image agreement (3.71 ± 0.38) and name agreement (0.79 ± 0.21) (Snodgrass and Vanderwart, 1980; Zhang and Yang, 2003). Among 30 pairs, 18 were separated into three groups which were used as the learning materials, and the other 12 were used as fillers which were not to be learned. Six lists of learning sequence were compiled through Latin-square to make sure that every group of learning materials can be learned under three different emotional states with equal opportunity. Participants were randomly administered to one of the lists.

Emotional pictures selected from the International Affective Picture System (IAPS, Lang et al., 1999) and Chinese Affective Picture System (CAPS, Bai et al., 2005) were used as the emotional state inducing materials which were divided into three groups. In each group, there were 144 pictures with positive, negative or neutral affective information. Emotional pictures were rated for valence from 1 (extremely negative) to 9 (extremely positive) and for arousal from 1 (extremely calming) to 9 (extremely exciting). As showed in Table 1, the emotional valence of pictures was significantly different among three groups (*p*s < 0.001) while there was no significant difference in the arousal level between negative and positive groups.

**Table 1:** Means (SDs) of parameters for three types of pictures.

	**Valence**	**Arousal**
Negative	2.04 (1.40)	6.04 (2.40)
Positive	7.25 (1.61)	5.69 (2.25)
Neutral	4.81 (1.55)	3.62 (1.95)

#### Procedure

Firstly, participants familiarized themselves with the form of the Korean characters through a word form judgment task in which they judged if the successively presented Hangul words were the same or different. Three types of fonts for each character (in Batang, Dutom and Gulim) were included in order that participants paid more attention to the orthography of the characters rather than the physical attributes. The familiarization process ended when their task accuracy attained over 80%. After that, they practiced the learning procedure with extra stimuli which wouldn't be presented during the formal learning process.

##### Learning procedure

**Figure 1:**
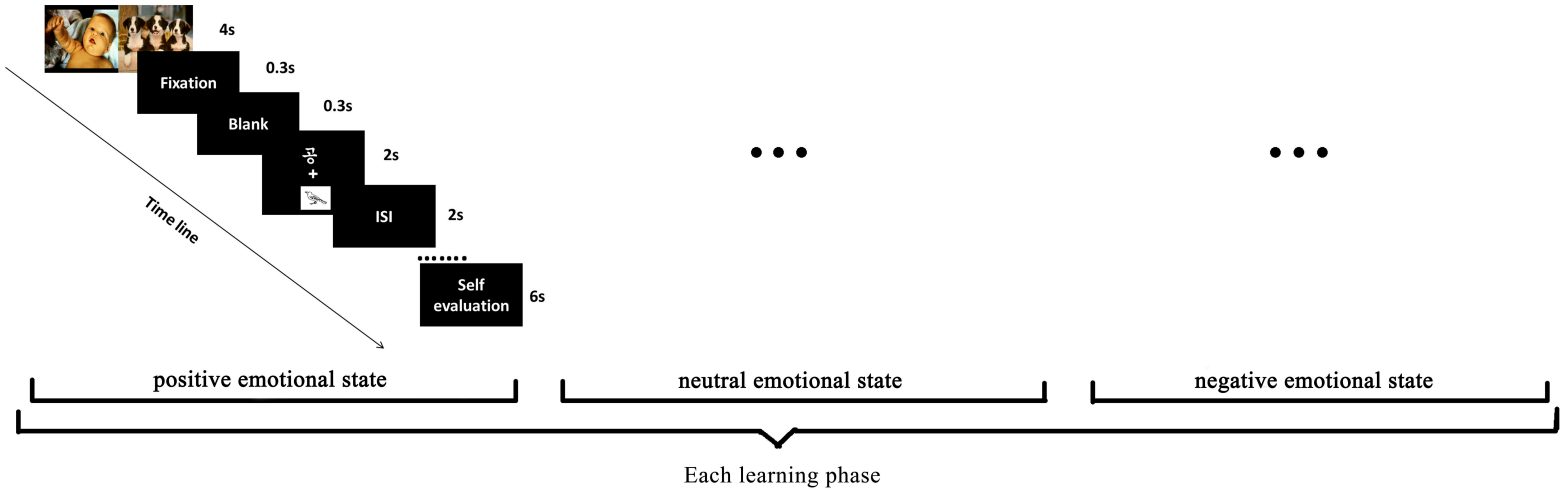
Learning procedure in each learning phase.

There were four learning phases in a row. We adopted the procedure containing alternative blocks of emotion induction (EI) and learning trial (LT) used by Schmitz et al. (2009) in each learning phase. Three types of emotional states were arranged sequentially to obtain relatively sustained emotional experience, and the sequence of EIs in each learning phase was counter-balanced between participants. To ensure that emotional states are successfully induced and sustained during learning, we used two emotional pictures in each EI block with the comparable duration as LT block. As Figure 1 illustrated, two emotional pictures with like valence and arousal were consecutively presented for 2,000 ms each in an EI block, and followed by a LT block. In a specific LT block, a 300 ms fixation “+” appeared firstly, after a 300 ms blank, a white-colored Hangul word against black background was presented above the fixation for 2,000 ms and was accompanied by a correlated white and black line drawing which was presented below the fixation simultaneously. Participants were asked to associate the meaning of word with the drawing. After that, the Hangul word appeared at the middle of screen and participants were asked to judge if it's animate or inanimate through keyboard pressing. Inter-trial-interval was randomized from 500 to 1,000 ms. There were 18 cycles of EI and LT blocks for each emotional state so that all three fonts of the 6 Hangul words were learned once. To avoid the suppression of emotional arousal by labeling (e.g., Lieberman et al., 2011), measures for the emotional state manipulation were collected only after participants finished the learning under a specific emotional state. Then they would evaluate their subjective experience on a 9-point rating scale (1 means extremely negative, 9 means extremely positive) within 6 s. After that, they would go on with the word learning under another emotional state until all 18 novel words were learned. The arrangements were similar in the remaining three learning phases. However, to avoid the adaptation of the emotional pictures, each picture was used only once.

##### Testing procedure

Tests were administered after each learning phase, and participants were asked to perform the semantic category judgment task in which they judged if the Hangul words were animate or inanimate through keyboard pressing as soon and correctly as possible. All the learned words and unlearned words were included in the tests.

Since words' emotional information can affect the processing of the words *per se*, the effects of emotional states on word learning may be confused with the impact of emotional information of learned words if they catch some corresponding affective meaning during learning. To eliminate the potential confusions, we asked participants to evaluate their subjective preference for all the Hangul words again on a 9-point rating scale (1 means extremely dislike, 9 means extremely like) after four learning phases.

### Results and discussion

Data were analyzed through SPSS 16.0. Two participants were excluded due to failing to finish experiment or chance level accuracy during all the phases of learning, so there were 18 participants included in data analyses. The accuracy rate of task during learning is around 96.6% for all conditions, which meant the participants fulfilled the learning task as required.

#### Self-evaluation of emotional state

The self-evaluated scores of emotion state were shown in Table 2. The 3 (Emotional State, ES) by 4 (Learning Phase, LP) repeated ANOVAs were applied to the self-reported scores. Results showed that there was a significant ES effect [*F*_(2, 34)_ = 67.55, *p* < 0.001, η^2^ = 0.80]. Multiple comparisons revealed that the values for negative ES were significantly lower than those for neutral ES which were linearly lower than the values for positive ES. The main effect of learning phase and the interaction between LP and ES were not significant [*F*_(3, 51)_ < 1; *F*_(6, 102)_ = 1.04, *p* = 0.4]. These results suggested that emotional states were induced as expected and were kept stable across four learning phases.

**Table 2:** Means (SDs) of self-evaluated value of emotional state in Experiment 1.

**Emotional state**	**Learning phase 1**	**Learning phase 2**	**Learning phase 3**	**Learning phase 4**
Positive	6.28 (1.78)	6.38 (1.24)	6.22 (1.59)	6.78 (1.66)
Negative	3.33 (2.33)	2.89 (2.08)	2.72 (1.99)	2.72 (1.90)
Neutral	5.67 (1.37)	5.56 (1.29)	5.67 (0.84)	5.50 (1.04)

#### Preference to hangul words

The scores of preference to Hangul words in different learning conditions were: 6.04 (0.91) for learned words under negative ES, 6.12 (1.04) for learned words under neutral ES, 6.17 (0.97) for learned words under positive ES, and 4.77 (0.85) for unlearned words. One-way ANOVA analysis showed there were significant differences between learned and unlearned words [*F*_(3, 51)_ = 12.50, *p* < 0.001, η^2^ = 0.42]. Participants showed more preference to learned words than unlearned words; however, they didn't show different preference to the words learned under different emotional states, which meant the preference to learned words was probably due to the mere exposure effect rather than the learned words caught the affective meaning during learning (Zajonc, 1968).

#### Semantic category judgment

Before performing statistical analyses for response time (RT), we excluded the trials with incorrect responses and outliers which fell out the 2.5 standard deviations. The means and SDs of accuracy and RT were shown in Tables 3, 4. As the tables showed, the accuracy for the learned words under all three types of emotion states increased while the RTs decreased with learning continuing; however, the accuracy for unlearned words stayed at chance level across four learning phases. These results confirmed that the changes of behavioral indices during tests were due to the acquisition of learned words other than the familiarity of testing tasks.

**Table 3:** Means (SDs) of ACC in semantic category judgment task.

**Emotional state**	**Learning phase 1**	**Learning phase 2**	**Learning phase 3**	**Learning phase 4**
Negative	0.60 (0.15)	0.66 (0.15)	0.85 (0.13)	0.89 (0.12)
Positive	0.67 (0.12)	0.78 (0.18)	0.86 (0.11)	0.89 (0.13)
Neutral	0.55 (0.15)	0.80 (0.12)	0.86 (0.13)	0.92 (0.07)
unlearned	0.48 (0.12)	0.51 (0.12)	0.53 (0.15)	0.47 (0.11)

**Table 4:** Means (SDs) of RT in semantic category judgment task.

**Emotional state**	**Learning phase 1**	**Learning phase 2**	**Learning phase 3**	**Learning phase 4**
Negative	1,235.32 (642)	1,199.61 (410)	1,088.88 (311)	992.66 (251)
Positive	1,218.53 (530)	1,221.33 (316)	1,090.48 (287)	972.07 (301)
Neutral	1,188.35 (513)	1,322.66 (474)	1,139.39 (396)	1,022.68 (291)
unlearned	1,404.48 (737)	1,559.39 (507)	1,469.07 (568)	1,219.24 (364)

The 3 (Emotional State, ES) ^*^ 4 (Learning Phase, LP) repeated ANOVAs were conducted on the mean accuracy and RT (To be more concise, the unlearned trials were not included in statistics). For the mean accuracy, the main effect of learning phase was significant [*F*_(3, 51)_ = 61.93, *p* < 0.001, η^2^ = 0.79], and that of ES was not [*F*_(2, 34)_ = 2.16, *p* = 0.13, η^2^ = 0.11]. Importantly, there was significant interaction between ES and LP [*F*_(6, 102)_ = 3.88, *p* < 0.02, η^2^ = 0.19]. The simple effect analysis revealed that the ES effect mainly existed in the first [*F*_(2, 34)_ = 4.27, *p* < 0.02, η^2^ = 0.20] and second [*F*_(2, 34)_ = 5.65, *p* < 0.008, η^2^ = 0.25] learning phase. *Post-hoc* analysis revealed that the accuracy under positive ES were higher than that under neutral and negative ES in the first learning phase, and in the second learning phase, the mean accuracy under negative ES was significantly lower than that under neutral and positive ES. These results suggested that the effects of emotional state were mainly phenomenal at the beginning of learning. Specifically, positive emotional state may enhance the learning process, but the negative emotional state may hinder the learning process.

For the RTs, the main effect of LP was significant [*F*_(3, 51)_ = 5.13, *p* < 0.05, η^2^ = 0.23]. However, the main effect of emotional state and the interaction between ES and LP were not significant [*F*_(2, 34)_ < 1; *F*_(6, 102)_ < 1].

Taken together, results of Experiment 1 revealed that the emotional states could exert significant influence on later retrieval of semantic category information. More importantly, the impact of emotion, whether enhancement effect from positive emotional state or impediment effect from negative emotional state, mainly existed at the beginning of learning, and disappeared at the end of learning. The eventual accuracy of retrieval of sematic category was around 90% for all words learned under three kinds of emotional states, which meant participants could attain high level proficiency through repeated learning even if their emotional state during learning may impact learning efficiency. Compared to the words learned under neutral condition, the participants' preference for words learned under positive and negative condition did not show significant difference. In other words, learned words did not catch the specific affective meaning after learning. Therefore, the emotional state during learning impacts the learning efficiency but not the preference for words.

It was noted that participants were only asked to grasp the categorical information of the Hangul words in this experiment, i.e., they just needed to know if a Hangul word was animate or inanimate to fulfill the learning task rather than to obtain the specific one to one mapping of a Hangul word with a specific concept. Categorical information is the general and basic concept of a word, based on which participants can further acquire the specific meaning of a word. Experiment 1 suggested that emotional states could impact novel words' categorical information learning in the first and second learning phases, but if the regulating effect of emotions would be shifted when participants have to learn more detailed information of the novel words? The following experiment aimed to settle this question.

## Experiment 2: emotional modulation on novel words' specific meaning learning

### Methods

#### Participants

Twenty-three right-handed Chinese students (9 males, average age was 22) from Beijing Normal University participated in this experiment with a little amount of money as payment. All participants have normal or corrected to normal eyesight and they are screened by PANAS before experiments to ensure their emotional stability.

#### Materials

The materials were the same with that used in the Experiment 1.

#### Procedure

The practice and learning processes were similar to the Experiment 1. Compared to Experiment 1, the goal or instructions of learning changed into mastering the specific meanings of the words instead of basic semantic categorical information. Therefore, participants were asked to fulfill word-picture semantic consistency judgment task during the testing phases. Specifically, participants judged if the simultaneously presented Hangul words (above fixation) and pictures (below fixation) had the same meaning through keyboard pressing as soon and correctly as possible. If they were the same, press “F” or “J” button, otherwise hit “J” or “F.” The mapping relations between keyboard pressing and responses were counterbalanced between subjects. All the learned words and unlearned words were contained in the tests.

### Results and discussion

All data were analyzed using SPSS 16.0. Two participants' data were excluded due to their testing scores were around chance level through all four learning phases, which meant they failed to concentrate on the learning tasks. Another 3 participants' data were also deleted because their emotional states were not induced successfully by emotional pictures according to their performance on self-evaluation of emotional state after finishing learning under a specific emotional state. Therefore, there were 18 participants' data entering into the statistical analyses.

#### Self-evaluation of emotional states

The self-evaluated values of emotion state were shown in Table 5. The 3 (Emotional State, ES) by 4 (Learning Phase, LP) repeated ANOVAs were applied. Results showed that there was a significant ES effect [*F*_(2, 34)_ = 41.61, *p* < 0.001, η^2^ = 0.71]. Multiple comparisons revealed that the self-evaluated value for negative ES was significantly lower than that for neutral ES which is linearly lower than the value for positive ES. The main effect of learning phase and the interaction between LP and ES were not significant [*F*_(3, 51)_ = 1.09, *p* > 0.3, *F*_(6, 102)_ = 1.62, *p* = 0.1]. These results suggested that emotional states were elicited as expected and were kept stable across four learning phases.

**Table 5:** Means (SDs) of self-evaluated value of emotional state in Experiment 2.

**Emotional state**	**Learning phase 1**	**Learning phase 2**	**Learning phase 3**	**Learning phase 4**
Positive	6.72 (1.41)	5.72 (1.27)	5.67 (1.68)	6.05 (1.25)
Negative	2.78 (1.51)	2.56 (1.34)	3.00 (1.71)	2.94 (1.39)
Neutral	5.89 (1.45)	5.27 (0.83)	5.22 (0.88)	5.50 (1.50)

#### Preference to hangul words

The scores of preference to Hangul words in different learning conditions were: 5.85 (1.07) for learned words under negative ES, 5.91 (1.03) for learned words under neutral ES, 6.21 (1.28) for learned words under positive ES, and 4.69 (0.74) for unlearned words. One-way ANOVA analysis showed there was significant difference between learned and unlearned words [*F*_(3, 51)_ = 10.57, *p* < 0.001, η^2^ = 0.38]. *Post-hoc* analysis showed that the preference to learned words was significantly higher than unlearned words, but the preferences for the learned words under different emotional states were not different.

#### Word-picture semantic consistency judgment task

Before statistical analyses for response time (RT), we excluded the trials with incorrect responses and outliers which fell out the 2.5 standard deviations. The means and SDs of accuracy and RT were shown in Tables 6, 7. As the tables suggested, the accuracy for the learned words increased and the RTs decreased with learning continuing; however, the accuracy for the unlearned words stayed at chance level across four learning phases.

**Table 6:** Means (SDs) of ACC in word-picture semantic consistency judgment task.

**Emotional state**	**Learning phase 1**	**Learning phase 2**	**Learning phase 3**	**Learning phase 4**
Negative	0.58 (0.18)	0.71 (0.13)	0.76 (0.13)	0.83 (0.11)
Positive	0.62 (0.15)	0.72 (0.11)	0.85 (0.10)	0.84 (0.12)
Neutral	0.57 (0.15)	0.77 (0.09)	0.82 (0.10)	0.84 (0.15)
unlearned	0.50 (0.11)	0.51 (0.08)	0.49 (0.11)	0.49 (0.09)

**Table 7:** Means (SDs) of RT in word-picture semantic consistency judgment task.

**Emotional state**	**Learning phase 1**	**Learning phase 2**	**Learning phase 3**	**Learning phase 4**
Negative	1,543.37 (228)	1,513.37 (278)	1,377.51 (185)	1,286.70 (252)
Positive	1,470.82 (275)	1,464.74 (201)	1,358.20 (193)	1,301.43 (213)
Neutral	1,518.30 (246)	1,433.67 (252)	1,387.00 (252)	1,299.19 (191)
unlearned	1,564.30 (300)	1,406.55 (314)	1,316.33 (335)	1,258.69 (269)

The 3 (Emotional State, ES) ^*^ 4 (Learning Phase, LP) repeated ANOVAs were conducted on the mean accuracy and RT. For the mean accuracy, the main effect of LP and the main effect of ES were significant [*F*_(3, 51)_ = 46.89, *p* < 0.0001, η^2^ = 0.73; *F*_(2, 34)_ = 3.74, *p* < 0.05, η^2^ = 0.18]. Furthermore, the interaction between ES and LP was significant [*F*_(6, 102)_ = 5.76, *p* < 0.05, η^2^ = 0.08]. The simple effect analysis revealed that the ES effect mainly existed in the second [*F*_(2, 34)_ = 3.22, *p* < 0.052, η^2^ = 0.16] and third [*F*_(2, 34)_ = 7.35, *p* < 0.02, η^2^ = 0.3] learning phase. *Post-hoc* analysis revealed that the accuracy under negative ES and positive ES were lower than that under neutral ES in the second learning phase, and there was no difference between positive and negative ES. While in the third learning phase, the accuracy under negative ES were significantly lower than that under neutral and positive ES, and the difference between neutral and positive ES disappeared. These results suggested that the effects of emotional state were relatively prolonged to mid learning phases compared to the findings of Experiment 1.

For the RTs, the main effect of LP was significant [*F*_(3, 51)_ = 12.43, *p* < 0.001]. However, the main effect of ES and the interaction between LP and ES were not significant [*F*_(2, 34)_ < 1; *F*_(6, 102)_ = 1.06, *p* > 0.3].

Results of Experiment 2 basically replicated the first experiment's findings except for that the effect of emotional state appeared in later learning phases. In accord with Experiment 1, negative emotional state impeded the specific words' meaning retrieval in the second and third learning phases, but the emotional lagging effects could be offset through continued learning or practice. However, compared to neutral emotional state, positive emotional state showed mild negative effect on novel words' specific meaning retrieval in the second learning phase rather than enhancing effect on words' categorical information retrieval. In other words, the effect of positive emotion depends on learning task or depth. In addition, Experiment 2 squared with the findings that learned words could not catch the specific affective meaning although they were acquired in emotional environments. Therefore, it was not the affective meaning of learned words but the emotional state during learning that impacted the learning efficiency.

## General discussion

The present study assessed the influence of emotional states on novel word learning by having participants perform a semantic category learning task or word-picture semantic consistency learning task. As we expected, positive and negative emotions exerted different impacts on novel word learning. Negative emotional state led to worse learning performance relative to positive and neutral emotional states, which paralleled previous research studying the interplay of emotions and cognitive processes (Isen et al., 1987). Positive emotional state showed a facilitating effect in semantic category learning but did not in semantic specific meaning learning. More importantly, the effects of emotional states were not unchangeable across four learning phases, and they were more dramatic during the early phases of learning. Moreover, different tasks which indicated different depths of novel word learning moderated the emotional effects.

The learning effects were significant in both experiments as the accuracy for the learned words increased and the RTs decreased with learning continuing. This is not surprising because the ability to acquire novel words from a limited number of exposures is one of the most remarkable capacities of the human brain (Takashima et al., 2014). For instance, young children can associate novel word-forms with meaning extracted from context extremely rapidly, after only a single or very few encounters with a novel word (“fast mapping”; Carey and Bartlett, 1978). Although word-picture semantic consistency learning task is more difficult compared to semantic category learning task which can be manifested by the lower accuracy and longer RTs, participants still showed a dramatic learning curve.

Suppressing effect of negative emotional state was repeatable in both our experiments. Such results might result from the attention allocation during learning. Focusing attention on materials is the first requirement for successful learning (Knörzer et al., 2016). Emotions affect a learner's attention by altering the scope of attention (Gasper and Clore, 2002; Fredrickson and Branigan, 2005; Rowe et al., 2007; Schmitz et al., 2009; Uddenberg and Shim, 2015), and studies have found that a narrowed attention focus often occurred with negative emotions (Fredrickson, 2000; Kaspar and König, 2012). Additionally, irrelevant thoughts induced by negative emotions can distract learners from the study materials (Seibert and Ellis, 1991), indicating that emotions interfere with optimal attention allocation on the materials and thus result in worse learning performance. In the present study, emotional states were induced by emotional pictures before learning trials, and negative emotional arousal led to narrower scope of attention thus impacted the mapping of word orthography and semantics. According to Zeng et al. (2016), the threat level of the emotion-laden stimulus is crucial to determining the facilitating or suppressing effect on task performance if the emotion-laden stimulus is task-irrelevant. The emotion-laden stimulus of a high threat will impair task performance as it competes with the perceptual processing. The emotion-laden stimulus present before learning in our study is task-irrelevant, so suppressing effect of negative emotions might be attributed to the high threat level of emotional pictures.

It should be noted that the suppressing effect of negative emotional state was inconsistent with a recent study by Knörzer et al. (2016) which observed a facilitating effect of an induced negative emotional state on learning outcomes. Nevertheless, several differences between their study and the present study might explain the opposing results. Firstly, the learning material in their study differed from ours regarding the type and structure of the knowledge. An established multimedia instruction about biology was used in the study of Knörzer et al. (2016), while Hangul words served as learning material in the present study. Secondly, there was task difference between the two studies. Unlike the present study, retention, comprehension, and transfer tests were administered in their study. So the difference of required cognitive processing might have an influence on the direction of effects. Thirdly, they used a combination of music and autobiographic recall to induce happiness (a positive activating emotion) or sadness (a negative deactivating emotion), while in the present study positive and negative emotional states with comparable arousal were induced by emotional pictures. The induction of different components of emotions may result in different effects on learning performance. In the field of emotional impact on learning, diverse emotion induction procedures were employed, such as videos (Plass et al., 2014; Morgan and D'mello, 2016), anthropomorphisms (Park et al., 2015), a combination of music and autobiographic recall (Knörzer et al., 2016), or positively or neutrally valenced statements (Um and Plass, 2012). In the present study, we used affective pictures in laboratory settings to induce learners' emotional states. However, to what extent everyday-life or more naturalistic emotions exert different influence on learning compared with experimental emotions, will need to be further tested applying various learning materials and paradigms in future studies.

Facilitating effects of positive emotional state occurred in the category learning but not in the specific meaning learning, which meant the effect of emotional state could be shifted by leaning depth as we expected. On the one hand, positive emotional state showed a facilitating effect in semantic category learning task. Positive emotions are expected to enhance motivation (Efklides et al., 2006) and therefore lead to better learning (Pekrun, 2006). Alternatively, the learner's optimism and confidence enhanced by positive emotions facilitate the activation of cognitive processes (Schwarz, 2000). On the other hand, the enhancing effect induced by positive emotional state disappeared in word specific meaning learning task. This finding can be explained by Cognitive Load Theory (Plass et al., 2011), which deems emotions as a source of extraneous cognitive load. According to this view, ample cognitive resources are not available for successful learning because they are taken up by emotion arousal (Plass and Kaplan, 2016). The inconsistent effects of positive emotional state on novel word learning were not contradictory, but reflected the important role of learning task in moderating the emotional effects. As the Yerkes-Dodson law (Yerkes and Dodson, 1908) suggested, the optimal level of arousal for a more difficult task will be lower than for an easier task (the “task difficulty” hypothesis), thereby the effects of the same level of arousal for different tasks could be different. In the present study, positive emotion induction enhanced the approach motivation, and facilitated the learning proficiency in the easier semantic category learning task. While in the difficult specific meaning learning task, the similar positive emotion induction probably exceeded the optimal arousal level and led to suppressive effects on learning proficiency.

To catch the dynamic changes of the effects of emotional states on learning process, we compared the effects of emotion states in four learning phases. The results showed that the effects of emotional states didn't remain unchangeable across four learning phases. The impacts of emotions were notable in the early phases of learning but did not last to the end. Importantly, the self-evaluation of the subjective emotional experience suggested that the emotional states were induced as expected and remained stable across four learning phases. Therefore, the offset of emotional impact could not be attributed to different level of emotional induction between the early and later learning phases. It seemed that with the familiarity to the to-be-learned words enhanced, participants could separate themselves from the emotional influence instead. However, there might be an alternative explanation for the results. The emotional responses elicited by emotional pictures in the later phases of learning might not as strong as participants' subjective ratings indicated due to the automatic emotional regulation. Therefore, the impact of emotion might be stronger in the early phases of learning and weakened then. Based on the findings of Nielson and Lorber (2009), the use of the emotional regulation strategy termed reappraisal (Gross, 1999) led to reduced susceptibility to memory modulation by arousal, with the mood and arousal state ratings unaffected by emotional regulation. In other words, the impact of emotional arousal on memory performance is not dependent upon subjective response to the arousal (Nielson and Meltzer, 2009), but it can be mediated by emotion regulation traits and a predisposition toward arousal (Nielson and Lorber, 2009).

However, it is noted that the main effect of emotional state and the interaction between emotional state and learning phase for RTs were not significant in both experiments. This might be due to the difficulty of the tasks requiring the participants to command completely novel words in a short time in our study, which entailed too much cognitive load to ensure the accuracy of tests, so that RTs were not sensitive to the tasks. Another possible reason is that the impact of emotions became weakened at the testing phases. Since emotions represent a short-term affective response and tend to decay quickly once its inducement is removed (Pérez et al., 2016), the emotional impact on lateral retrieval may not be evident for RTs.

There is a stronger and stronger emphasis on research examining the interplay between emotion and learning, especially the impact of emotion on multimedia learning (Um and Plass, 2012; Park et al., 2014, 2015; Plass et al., 2014; Knörzer et al., 2016). The emotional design hypothesis postulates that “making essential elements visually appealing initiates and guides cognitive processing during learning” (Plass et al., 2009; Mayer and Estrella, 2014). A proposed underlying mechanism for the impact of emotional design elements is that “they guide learners' attention and maintain cognitive processing by increasing motivation” (Park et al., 2015). Research in this field implicates suggestions for a sound instructional design of learning contexts which should take into account the learners' emotional states. The present results suggested a beneficial effect of positive emotional state for simpler learning tasks, in line with many studies which explored the difference between positive and negative affect over a broad range of tasks with positive affect found to be advantageous in selective tasks (e.g., Fredrickson, 2003; Fredrickson and Branigan, 2005; Lyubomirsky et al., 2005). Based on our findings, teachers and instructional designers should create learning environments that promote appropriate positive emotions, avoiding negative emotional states.

The current research has some limitations. Firstly, individual differences, such as, working memory capacity (Baddeley et al., 1998) and arousal predisposition (Nielson and Lorber, 2009), might serve as potential confounding variables. In terms of arousal predisposition, highly predisposed individuals have greater subjective and physiological responses to arousing stimuli (Coren and Mah, 1993), and therefore might be affected to different extents during learning. Secondly, although arousal is controlled in this study, it might still have influence on our findings. So there should be extended research to investigate the role of the activation dimension of emotions in novel word learning. Thirdly, wordlist studies may not provide enough ecological validity to assist in applying such approaches to classroom settings (Nielson and Arentsen, 2012), therefore future work could apply more authentic learning environment and relatively naturalistic emotional states. Last but not least, novel word learning in this study is a type of explicit associative learning and mainly focuses on the lexical orthography-semantic associative learning with phonology not emphasized. Hence, future research needs to employ a variety of paradigms to fully investigate the mechanism of novel word learning.

## Conclusion

The present study investigated the dynamic influence of emotional state on novel word learning. It was suggested that positive and negative emotional states exerted different influence on novel word learning. The induced positive emotional state facilitated successful learning only in a shallower semantic category learning task. Conversely, induced negative emotional state generally showed an overall suppressing effect on novel word learning. Secondly, the effect of emotional state mainly existed in the early phases of learning, and the responses to the learned words under different emotional conditions turned out to be similar at the end of learning. The present study could provide further insight into emotion-cognition interactions, and it will be enlightening to explore the impact of emotion on various depths of learning in future studies.

## Ethics statement

This study adhered to the Declaration of Helsinki and was approved by the Ethical Committee of Shaanxi Normal University. All participants gave written informed consent prior to the study.

## Author contributions

JG and DP conceptualized this study; JG contributed to the design and implementation of data collection; JG and TZ drafted the manuscript.

### Conflict of interest statement

The authors declare that the research was conducted in the absence of any commercial or financial relationships that could be construed as a potential conflict of interest. The reviewer CD, and handling Editor declared their shared affiliation.

## Appendix A

**Table A1:** Examples of hangul words.

**Number**	**Hangul**	**Strokes**	**Radical**	**Number**	**Hangul**	**Strokes**	**Radical**
**1**	또	6	3	**16**	습	7	3
**2**	통	7	2	**17**	못	7	2
**3**	생	7	3	**18**	잘	8	3
**4**	체	7	2	**19**	백	8	3
**5**	림	7	3	**20**	감	6	3
**6**	면	7	3	**21**	축	6	2
**7**	집	7	3	**22**	문	6	3
**8**	밖	9	3	**23**	육	7	2
**9**	멈	8	3	**24**	효	7	3
**10**	끝	8	2	**25**	퓨	7	2
**11**	부	6	2	**26**	락	6	3
**12**	들	6	3	**27**	회	7	2
**13**	원	7	3	**28**	분	7	3
**14**	렁	7	3	**29**	름	8	3
**15**	쪽	7	2	**30**	쓸	8	3
